# Interaction of Environmental Pollutants with Microplastics: A Critical Review of Sorption Factors, Bioaccumulation and Ecotoxicological Effects

**DOI:** 10.3390/toxics8020040

**Published:** 2020-06-02

**Authors:** Albert Menéndez-Pedriza, Joaquim Jaumot

**Affiliations:** Department of Environmental Chemistry, IDAEA-CSIC, Jordi Girona 18-26, 08034 Barcelona, Spain; ampqam@idaea.csic.es

**Keywords:** microplastics, environmental pollutants, sorption, bioaccumulation, toxicological studies

## Abstract

Microplastics have become one of the leading environmental threats due to their persistence, ubiquity and intrinsic toxic potential. The potential harm that microplastics impose on ecosystems varies from direct effects (i.e., entanglement and ingestion) to their ability to sorb a diversity of environmental pollutants (e.g., heavy metals, persistent organic compounds or pharmaceuticals). Therefore, the toxicological assessment of the combined effects of microplastics and sorbed pollutants can produce in biota is one of the hottest topics on the environmental toxicology field. This review aims to clarify the main impacts that this interaction could have on ecosystems by (1) highlighting the principal factors that influence the microplastics sorption capacities; (2) discussing the potential scenarios in which microplastics may have an essential role on the bioaccumulation and transfer of chemicals; and (3) reviewing the recently published studies describing toxicological effects caused by the combination of microplastics and their sorbed chemicals. Finally, a discussion regarding the need for a new generation of toxicological studies is presented.

## 1. Introduction

The term plastic is a generic name encompassing most of the synthetic and semisynthetic organic polymers which are capable of exhibit plasticity. These materials are ideal for a large variety of applications due to their versatility, durability, lightweight, chemically inert behaviour and their low-cost-production, among others. All these outstanding features could explain why global plastic production has increased significantly over the past decades (1.7 million tons in the 1950s to 359 million tons in 2018, Figure 1a) [1]. Moreover, experts believe that consumer needs will not decline in the near future. Consequently, by 2050, the world’s plastic production is estimated to exceed the 1000 million metric tons per year. Although the societal benefits of plastic materials are indubitable, the poor management in plastic debris through the last decades has led plastics to currently become one of the largest portions of the municipal waste. Until the year 2017, estimates stated that approximately 6300 million tons of plastic waste had been generated globally. From this huge amount, only twenty per cent of this litter was recycled or incinerated. In contrast, the remaining eighty per cent was either accumulated in landfills or released to natural environments (e.g., marine and freshwater ecosystems) [2]. These estimates also affirmed that around ten per cent of the plastic produced over the years had been discharged in the marine environment. All these factors lead to the fact that plastic debris already encompasses for sixty to eighty per cent of the marine litter, after less than a century of existence [3]. Unfortunately, these effects could be aggravating in the near future as estimates indicate that roughly eight million tons of plastic materials end up in the marine environment every year [4]. Hence, plastic marine litter has become a global environmental menace due to its persistence, ubiquity and toxic potential. Recent studies have also raised concerns on the ability of microplastics (MPs) to sorb on their surface a vast number of environmental compounds (e.g., heavy metals, persistent organic compounds (POPs), pharmaceuticals) [5,6,7,8]. Thus, considering the new shreds of evidence on the multiple risks that plastics pose to the environment, different marine protection projects such as the Marine Debris Program of the US National Oceanographic and Atmospheric Administration (NOAA), included plastics litter as an emerging pollutant [9]. Therefore, the evaluation of the possible ecological impacts of plastic waste in marine ecosystems has currently become one of the key research fields for the scientific community, highlighting the particular attention posed on small pieces of plastic, referred to as MPs.

Small pieces of floating plastics in the ocean surface were firstly reported in the scientific literature in the early 1970s. However, the NOAA did not define MPs as synthetic polymers with an upper size limit of 5 mm until 2009 [10]. MPs are divided into large (1–5 mm) and small (1–1000 µm). Larger plastics were categorized as megaplastics (larger than 1000 mm), macroplastics (from 250 to 1000 mm) and mesoplastics (from 5 to 250 mm). Below the 1 μm scale, plastics should be designated as nanoplastics (NPs), another rather unknown part of the marine waste. These NPs may also represent a risk in the environment due to their sizeable amount, but in-depth studies are required to understand their toxic effects and mechanisms.

**Figure 1:**
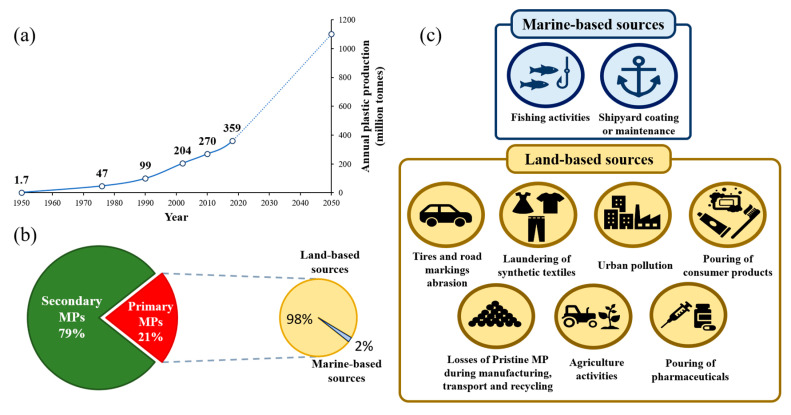
(**a**) Global plastic production since the 1950s together with an estimate of the global plastic production until 2050. (**b**) Estimated percentage of primary microplastics (MPs) that ends up into oceans respect the total MPs materials released into marine ecosystems every year. The percentage of land-based and marine-based contributions are also presented. (**c**) Most important land-based and marine-based sources of primary MPs released into marine ecosystems.

MPs can be classified into primary and secondary MPs. Primary MPs are manufactured plastics for industrial or domestic use of microscopic scale. Virgin or pristine plastic pellets are also considered as primary MPs. There are several applications of these primary MPs in various industries [11,12] such as cosmetics (i.e., facial cleansers, toothpaste or shower gels), textile (i.e., stockings, faux leather, fur or suits) and medical applications (i.e., vectors for drugs). Besides, the large amounts of primary MPs generated in the car tires abrasion while driving and the laundering of synthetic textiles make them the main sources of generation of this type of MPs. Therefore, land-based activities produce almost all the losses of primary MPs (Figure 1b) [13]. However, these primary MPs commonly end up in either fresh-water or seawater environments through different pathways such as wind transfer, discharge from wastewater plants systems, road runoff and industrial or domestic drainage. All these contributions result in an estimated global release of between 0.8 and 2.5 Mtons of primary MPs into the marine environment every year according to an optimistic or pessimistic scenario, respectively (Figure 1b) [13]. The following example is introduced to highlight this contribution. Considering a release of 1.53 Mtons/year of primary MPs, 43 light plastic bags should be thrown into oceans per person each week. Consequently, the proportion of primary MPs represent between 15% and 31% of the total MPs in the oceans according to the study by Boucher [13].

The plastic waste in the land and oceans can be further fragmented into much smaller particles due to degradation processes, which are more reactive and dangerous to marine fauna and humans. These MPs are known as secondary MPs and present a variety of origins, including fishing nets, industrial resin pellets, household items, and other discarded plastic debris [12]. A variety of environmental and mechanical factors control plastics fragmentation rates. Therefore, these environmentally-linked degradation processes can be classified as biodegradation (i.e., the action of living organisms), photodegradation (i.e., light radiation), thermo-oxidative degradation (i.e., slow oxidative breakdown at moderate temperature), thermal degradation (i.e., high temperature) and hydrolysis (i.e., water) [14]. It is established that land-based sources contribute to the formation of more than eighty per cent of the total microplastic debris in the marine environment [11]. This fact might be explained because these main degradation processes are more effective in land-based sources than in the aquatic environment. For instance, the UV radiation degradation mechanism (which commonly starts with a photooxidative degradation step) is more effective in plastics exposed in land-based sources (e.g., beaches). Photodegradation is also less efficient when the plastic material is floating in the water. The low temperature and oxygen concentrations in the aquatic environment may explain this decrease in the degradation efficiency [14]. However, the complete understanding of these degradation processes needs to combine the effects of several environmental factors and properties of the plastic polymers.

Due to the action of different natural processes such as infiltration, river discharge, wind, ocean currents, and the movement of animals and humans within and between ecosystems, MPs are in almost every habitat around the world (even in the polar regions [15]). In the aquatic ecosystems, MPs are present in every state of the water column (i.e., from surface waters to benthic zones). The nature and inherent properties of MPs (like shape, density or size) significantly influence their distribution, as well as the localization of the MPs sources and their subsequent complex interaction between physical, chemical and biological processes.

Currently, there is growing information about all these aspects related to the MPs effects on the environment. Many studies deal with the abundance and composition of MPs [16,17,18], but major uncertainties remain regarding the spatial and temporal distribution of MPs. Temporal variations and the lack of standardized analytical methods may be the main factors that explain this fact.

The MPs threat on ecosystems, marine organisms or humans due to their persistence and ubiquity explains the growing interest of the scientific community in this type of plastic debris [19]. These MPs’ environmental impacts can be classified in physical, biological and chemical effects.

Physical impacts include the entanglement and the ingestion of MPs, with entanglement being the most common, particularly for larger plastics (macroplastics and mesoplastics). However, the impacts of MPs on small-sized animals have been recently described in the literature. For instance, the entanglement of MPs to the swimming appendages of mysids was observed in the work of Lehtimieni [20]. Besides, large filter-feeding marine organisms may potentially ingest huge amounts of MPs due during feeding intervals. This phenomenon is especially significant in areas where zooplankton blooms concur with a higher accumulation of MPs (e.g., coastlines, thermal currents or ocean gyres such as the North Pacific central gyre) [21,22].

Ingestion of plastics can be direct or indirect. Direct ingestion occurs when animals eat them accidentally. In contrast, indirect ingestion is related to the trophic transfer being the result of the consumption of contaminated food. Several works document the ingestion of MPs at every trophic level, including zooplankton [23,24,25], mussels [26,27], fishes [25,28,29,30,31], sea turtles [32] and marine birds [25,33,34,35]. These physical impacts may induce drowning, suffocation, strangulation, and starvation [36] in addition to the damage to gills and other internal organs. Additionally, other effects such as reduction of the predatory performance, changes in the metabolism or the endocrine function, and other adverse effects potentially leading to death have also been reported [37,38,39,40,41]. Consequently, the lethal physical impact of plastic materials by marine animals has increased by almost 40% in the last decade, according to the Convention on Biological Diversity report [36].

Biological effects are caused by the changes in MPs physical properties due to the attachment of biofilms on their surface. Biofilms are phylogenetically and functionally diverse communities of bacteria, protozoans, algae, and fungi collectively forming a microbial assemblage, biofouling community, or periphyton [42]. Nowadays, the influence of such biofilms on the fate and potential effects of MPs is not well understood, and further investigation is needed [42,43]. Moreover, biological impacts also include the capacity to transfer microorganisms geographically.

Finally, the chemical impact of MPs might be attributed to residual monomers from manufacture present in the plastic. Furthermore, plastics may incorporate some chemical additives added during their production to improve physical properties such as colour, density, resistance or hardness. Many of these additives induce relevant ecotoxicological effects on humans and marine organisms. Examples of these additives are Bisphenol A (BPA) [44,45] (i.e., provides antioxidant properties to the plastic even though it is mainly found in the environment as a residual compound during the synthesis of polycarbonates and epoxy resins), several common-used flame retardants [46,47] and antimicrobial agents. [48]. Besides, the toxicity of some intermediates from partial degradation of plastics should also be considered [14]. However, recent studies have raised concerns on chemical effects due to the ability of MPs to sorb on their surface environmental pollutants. Examples of compounds sorbed to MPs surface are heavy metals or highly hydrophobic contaminants like persistent organic pollutants (POPs), including polychlorinated biphenyl (PCBs), polybrominated diphenyl ethers (PBDEs), dichlorobiphenyl trichloroethane (DDTs), hexachlorocyclohexanes (HCHs) and polycyclic aromatic hydrocarbon (PAHs) [49,50,51,52,53,54]. These studies pointed out a substantial enrichment of POPs in the polymers, often exceeding 10^6^ times relative to their concentrations in solution [55]. Similarly, the potential interaction of MPs with other emerging pollutants such as pharmaceuticals active compounds (PhACs), antibiotics, or UV filters have also raised the interest of the scientific community [56,57,58].

Therefore, MPs may play a crucial role in aquatic ecotoxicology acting as vectors for these highly toxic pollutants, becoming a potential source of lipophilic chemicals for bioaccumulation and biomagnification by facilitating their entrance to the food chain [59]. For that reason, several studies have assessed the potential bioaccumulation and bioavailability enhancement of chemicals previously sorbed on MPs [60,61,62,63,64]. Conversely, many authors have refuted the idea that MPs might have a relevant role in this bioaccumulation and biomagnification of toxic chemicals like POPs by marine animals. These works argue that plastic debris present in the oceans is still not enough to outcompete the partitioning of POPs to water and dissolved organic matter (DOM) [55,65,66]. Moreover, they highlight the unrealistic high concentrations used during most of the experiments, in addition to the incomparable sampling and analytical methods applied. For these reasons, the influence in the transport and bioaccumulation of pollutants by plastics is a current topic of debate within the scientific community. In the last years, several toxicological studies about MPs focused on the ecotoxicological assessment of their combined effects with environmental pollutants in diverse aquatic animals along with the food web. These studies are crucial to better understand the possible existence of interactions between plastic debris and these environmental pollutants as well as to assess the real toxicological impact that MPs have in the marine ecosystems.

To date, reviews on the behaviour of MPs in the environment have focused on summarizing their properties, sources, fate and occurrence [11,14,67], instrumental methods for their analysis [4,68], biological effects on organisms [19,36,69,70,71] and their chemical sorption capacities [72,73,74,75,76]. Nonetheless, the discussion about if MPs may act as a vector for POPs and other environmental pollutants, like heavy metals and pharmaceuticals, is still active. However, there is a lack of critical evaluation of the current research trends related to ecotoxicological effects assessed by the MPs chemical interaction in aquatic animals. Thus, this review aims to (i) describe the main factors influencing the sorption properties of plastics; (ii) present the different scenarios in which MPs may have an impact on the transfer and bioaccumulation of chemicals; and (iii) summarize the main ecotoxicological effects of MPs combined with sorbed environmental pollutants.

## 2. Factors Influencing the Sorption of Environmental Pollutants to MPs

The process of chemical transfer from a fluid phase (e.g., air or water) to a solid phase (e.g., plastic debris or DOM) defines the sorption of a compound. This term is associated with two types of processes: absorption and adsorption. The term absorption refers to the chemical interaction between compounds and a sorbent through relatively weak van-der-Waals forces in which molecules penetrate and become embedded within the matrix sorbent. In this phenomenon, the resulting partition coefficient (K_pw_) between plastic and water can be related to the octanol-to-water partition ratio (K_ow_) of polymers, in particular for the case of polyolefins [73,77]. In contrast, the term adsorption involves a variety of forces, from van-der-Waals to ionic, steric or covalent interactions. In these chemical processes, molecules remain in the interface between the fluid and the solid surface. In many cases, both absorption and adsorption processes may arise concurrently, so it is difficult to discriminate one interaction from the other [72]. However, adsorption processes predominate in the most usual scenario of low environmental concentrations of organic chemicals due to the stronger interactions with the solid phase surface. Otherwise, at higher concentrations, absorption is more likely to occur due to the larger amount of available compounds [74].

**Figure 2:**
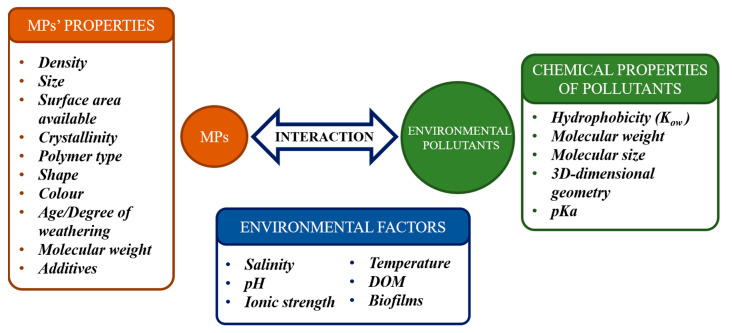
Summary of the main factors influencing the interaction between MPs and chemical pollutants.

Finally, it should be highlighted that these sorption processes are fundamentally related to the physicochemical properties of both the sorbate and the sorbent as well as the medium properties. Therefore, the sorption mechanisms between an organic pollutant and MPs depend on the interactions between them, which can be dominated by a specific contribution or even be a composed of several types of contributions. The most important factors to understand the pollutant-plastic interactions are graphically summarized in Figure 2 and will be described below.

### 2.1. Plastic Polymer Type

The inherent structural properties of a polymer (i.e., surface charge and area, molecular chain arrangement or acid-base character) are responsible for its fate. These attributes influence the sorption processes and the type of organic pollutants sorbed on the surface of the plastic particle. One of the most important plastic features affecting sorption processes is the degree of polymer crystallinity, which is related to the molecular chain arrangement. Polymers are composed both by crystalline and amorphous regions. Molecular segments showing a regular structure form the crystalline region, whereas areas with randomly packed chains constitute the amorphous region. In the structured domain, a high amount of energy is needed for the process of chemicals absorption. In contrast, random regions have a larger extent of free volume because of the distance between polymeric chains, which allows chemicals to diffuse more easily through the polymer. As a result of the size and complexity of these chains, polymers can only be semi-crystalline, mixing crystalline and amorphous regions, or completely amorphous [72]. Some examples of semi-crystalline polymers are polyethylene (PE), polypropylene (PP), polyethylene terephthalate (PET) and polytetrafluoroethylene (PTFE). Several factors affect polymer crystallinity, including polymer complexity, chain configuration, isomerism and rate of cooling during solidification.

**Figure 3:**
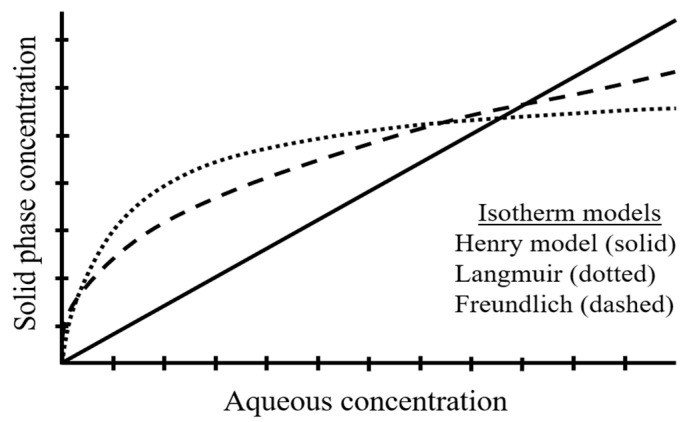
Standard isotherm models used in the study of sorption/desorption of environmental pollutants in MPs.

Another major characteristic to take into account is the glass transition temperature (T_g_), which is only related to the amorphous domains of the polymer. At temperatures below T_g_, the amorphous segments are in the glassy state, and above T_g_, they are in the rubbery state. It is crucial to mention that rubbery polymers (e.g., PE and PP) have higher diffusivity due to their larger free volume and their greater flexibility and mobility. Hence, these properties enhance the absorption of organic pollutants in these regions. The sorption isotherms of this type of polymers are essentially linear (i.e., Henry isotherm model, Figure 3), and the absorption process is reversible and non-competitive [78].

Conversely, glassy polymers (e.g., PS, PET and polyvinyl chloride (PVC)) are more condensed and present higher cohesive forces. Thereupon, this kind of polymers have long-lived and closed internal nanoscale pores which act as adsorption sites. For that reason, glassy polymers are responsible for lower release rates, as this trait creates stronger adsorption sites to organic compounds [74]. Consequently, adsorption processes may be the predominant sorption mechanism for glassy polymers [53]. Adsorption processes can be described applying several nonlinear isotherms (i.e., Langmuir and Freundlich nonlinear isotherm models, which are the most commonly used, Figure 3) and show competition mechanisms with any coexisting chemical [54,79]. Despite that, it must be considered that both absorption and adsorption processes may arise concurrently. Therefore, as pointed out in different studies, the Freundlich model is widely used to describe the pollutants-MPs interaction as enables a proper fitting of the results for many sorption/desorption processes [80,81,82].

Previous studies reported PE as the polymer, which sorbs and concentrates the highest amount of organic pollutants in comparison to PP and PVC [51,52,54]. Consequently, the order in the sorption capability of the most common plastic types has been determined by the following list: LDPE ≈ HDPE > PP > PVC ≈ PS [83]. PVC and PS are glassy polymers resulting in low mobility and low diffusivity of the sorbate. Despite that, other polymer characteristics can overrule the crystallinity effect, making its influence negligible. For example, Rochman et al. [50] reported that PS (glassy polymer) presents similar sorption capacities than LDPE and HDPE (rubbery polymers) for different PAHs. In this case, the authors argued that the greater segmental mobility of PE was compensated by the greater distance between polymeric chains in PS. Moreover, PS could undergo π-π interactions. Similar conclusions were reached by Seidensticker et al. (2018) [84]. This work demonstrated that the larger porous size of PS compared to PE enables a major sorption capacity of PS respect PE. Hence, these observations imply that the structural characteristics of each polymer have a profound influence on the sorption capacities of organic chemicals by plastics.

### 2.2. Size of the Plastic Pellets

Particle size is another relevant plastic property to understand the MPs-chemicals interaction since the sorption capacity is inversely proportional to the particle size (i.e., decreased particle size increases the surface area to volume ratio). For this reason, different authors underline their potential “Trojan horse” effect (i.e., hiding of the actual toxic potential of MPs and NPs due to their ability of sorbing multiple harmful pollutants) [8,60,85]. When sorption between MPs and NPs are compared, the sorption rates for NPs are one or two orders of magnitude higher [74]. However, this plastic size mainly affects adsorption rather than absorption, as the second does not depend on the availability of sorption sites on the surface [49]. This behaviour causes that authors need to be cautious regarding the influence of the particle size if the absorption process and other polymer features are involved. For instance, Hüffer and Hoffman [53] could not explain the sorption differences of a variety of organic chemicals with polyamide (PA), PE, PS and PVC considering only the MPs size effect and pointed out the considerable influence of hydrophobic interactions. It has to be also mentioned that we should also consider that the tendency for plastic particles to aggregate in the environment may lead to a reduction of the available surface area for chemical sorption. Consequently, particles’ aggregation may significantly affect the sorption capacities of plastics.

Summarizing, the size effect of plastic particles seems a matter of concern as smaller particles may represent a higher hazardous risk to the marine environment due to their increased potential to preconcentrate organic pollutants. Moreover, it also seems relevant to consider the actual size range distribution of plastic debris in the marine environment. In addition, the significance of processes involving MPs and NPs may vary depending on their relative abundance in the environment, and this could also influence the exposure perspective of the ecotoxicological studies evaluating their toxic potential.

### 2.3. Age and Degree of Weathering of Plastic

Pristine or virgin plastics are the newly manufactured and have not suffered any degradation by the environment. Conversely, aged or weathered plastics are those that have been exposed to degradation processes (i.e., thermal, mechanical, biological, radiative, oxidative breakdown or hydrolysis) [14,73]. The susceptibility to these environmental conditions enables the fragmentation and cracking of plastic debris into smaller particles. Therefore, an in-depth study of the degradation mechanisms of plastics caused by weathering, which facilitates the fragmentation of plastics into smaller-sized particles, is required.

Another important fact to point out is that aged pellets may also suffer chemical changes. For example, polymer crystallinity is susceptible to increase by weathering. For this reason, some authors considered that the sorption rates of organic pollutants by aged plastics might be reduced relative to the sorption rates of virgin plastics [86]. However, other studies reached opposite conclusions. For instance, Teuten et al. exposed that yellowing aged pellets had higher concentrations of PCBs [54]. Based on this finding, the International Pellet Watch project proposed a standardized methodology to monitor coastal pollution by hydrophobic chemicals [87]. They proposed analysing yellow pellets (i.e., the yellowing effect occurs as the result of phenolic oxidation agents to by-products with quinoidal structures that cause yellow colour), as these pellets tended to have higher concentrations of POPs. Results demonstrated the utility of weathered pellets to monitor POPs on a global scale. Accordingly, Chen et al. concluded that weathering enhanced the sorption capacity of different PAHs by MPs [88]. Other authors also confirmed that aged MPs lead to higher sorption capacities of POPs [52,89,90]. Chemical changes on aged plastics surface may also have significant relevance, as their surface can be oxidized, allowing the interaction with new hydrophilic organic compounds. Results noted by Huffer et al. pointed to this way, suggesting that the weathering process reduced the hydrophobicity of plastics due to the surface oxidation, which created new functional groups containing oxygen [91]. Similarly, Fu et al. reported that aged PVC micropellets were capable of sorbing higher amounts of copper due to surface changes induced by UV radiation [92]. Wang et al. revealed that the aged MPs had a higher adsorption capacity of heavy metals than pristine pellets. This fact was explained by the authors considering the correlation of the increased surface area and the oxygen-containing functional groups appeared in the surface of aged MPs after UV radiation [93]. Consequently, it seems that the functional groups and polarities of MPs significantly contribute to the accumulation of different types of pollutants from the environment.

Biological effects, for instance, the development of biofilms on the surface of plastic pellets, can also alter the polarity of these pellet surfaces as well as the specific surface-volume ratio [42]. Richard et al. concluded that biofilms enhanced the accumulation of various metals in plastic debris (e.g., Ga, Mn, Pb, Cu, Co, U, Fe, Ni, Al) [43]. In addition, Johansen et al. demonstrated that the enhanced sorption of Cs and Sr onto PE and PP MPs was possible due to the increased surface area allowed by the biofilms action [94].

**Figure 4:**
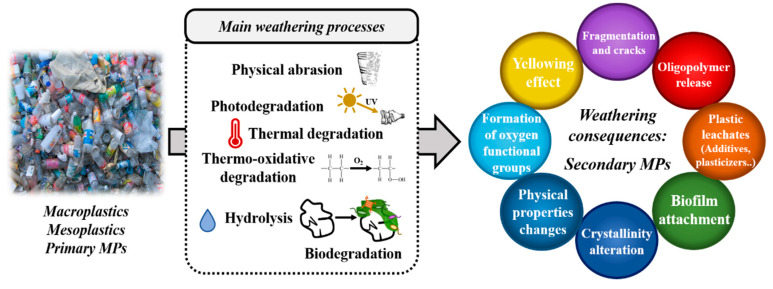
Overview of the main weathering processes and alterations produced on large plastic debris or primary MPs to form secondary MPs on marine environments.

Finally, it should be mentioned that weathering effects might also enable the release of toxic plasticizers and additives (e.g., phthalates, alkylphenols). Therefore, the weathering or ageing effect can change the sorption capacity of organic pollutants through the following aspects as represented in Figure 4: (1) fragmentation of larger plastic debris increases the specific surface area; (2) modulation of polymers properties (e.g., crystallinity); (3) oxygen-containing functional groups change surface properties on MPs by decreasing their hydrophobicity; and (4) attached biofilms improve the sorption capacity of MPs.

### 2.4. Chemical Properties of Pollutants

Chemical properties of organic pollutants are as relevant as polymer characteristics in determining their sorption rates to plastics. For organic chemicals, hydrophobicity and molecular weight are the most relevant attributes to explain their sorption capacities. The chemical sorption coefficients on MPs are generally related to their K_ow_ due to the hydrophobic nature of plastic surfaces. For instance, Huffer and Hoffman concluded that hydrophobicity interactions are the most important to predict chemical-plastic interaction. However, they underline that log K_ow_ was not a good descriptor for the sorption process [53]. In contrast, the molecular weight might become more relevant than K_ow_ if diffusion was the rate-limiting process. Similarly, molecular size could play a critical role in the sorption coefficient for hydrophilic contaminants [72].

Another chemical property that may have a significant impact on the MPs sorption rates is the pK_a_. pH has a considerable influence on the modulation of MPs-pollutant interactions. For that reason, pK_a_ may determine whether chemicals sorbed to MPs might be more prone to be released when external pH significantly varies. Consequently, pK_a_ could explain which environmental pollutants would be more likely to be desorbed under physiological conditions [57]. The three-dimensional geometry of the molecule could also influence their sorption rate. Planar molecules typically have higher sorption coefficients than non-planar molecules of similar hydrophobicity due to the stronger surface adsorption showed by planar molecules. This fact could be explained because planar chemicals can move closer to the plastic surface than bulkier non-planar molecules [49]. For instance, examples of planar organic compounds are PAHs and PCBs. These compounds present well-known toxicological effects (e.g., endocrine disruption, hepatotoxicity, immunotoxicity, congenital disabilities and induction of several enzymes) due to the high-affinity interaction with the aryl hydrocarbon receptor (AhR), a cellular protein [95]. These potential threats highlight the importance of performing an accurate evaluation of the toxicological consequences of the “Trojan horse” effect that plastic debris can handle, especially for MPs and NPs.

### 2.5. Environmental Factors

Surrounding environmental conditions also modulate the pollutant-plastic interaction. pH influence on the sorption rates depends on the specific chemical-plastic interaction due to chemical speciation. For instance, Wang et al. reported that a pH decrease would significantly affect the perfluorooctanesulfonate (PFOS) sorption by PS and PE, whereas perfluorooctanesulfonamide (PFOSA) adsorption was independent of this variation. This fact indicated that PFOS-plastic sorption mechanisms undergo through electrostatic interactions [96]. Similarly, Yu et al. concluded that pH had a relevant impact on the sorption capacity of tetrabromobisphenol A (TBBPA) on microplastic beads. In this case, a decrease of the pH led to an increment of the sorption rate of this flame-retardant compound [97]. In agreement with these results, Elizalde-Velázquez et al. exposed that the pattern of nonsteroidal anti-inflammatory drugs (NSAID) sorption on MPs exhibited a pronounced pH dependency due to the pH effect on the speciation of the compounds and the surface charge of the particle [98]. In contrast, Holmes et al. exposed that higher sorption was observed as the pH of the solution increased for metals producing cationic species (e.g., Pb^2+^, Cd ^2+^, Ni^2+^, Co^2+^). The authors suggested that the decline in the relative abundance of free ions could explain this experimental observation [82].

Moreover, other environmental factors such as ionic strength, salinity or the presence of DOM could also influence the sorption and desorption of chemicals from plastics on aquatic environments. The relevance of the ionic strength depends on the extent of the electrostatic interactions involved in the sorption/desorption mechanisms. For example, the study performed by Wang et al. exposed that the increase of the ionic strength only affected the sorption of PFOS, whereas PFOSA adsorption was independent. This difference indicated that PFOS sorption mechanisms to plastic undergo through electrostatic interactions [96].

Salinity influence on the sorption capacities of chemicals by plastics is critical as it enables the differentiation of freshwater and marine environments. In this case, an increased salinity can neutralize the surface charges upon MPs via the compression of the electric double layer, which lowers the role of electrostatic interactions in the sorption of MPs. The high salinity also induces the salting-out effect of chemicals and, consequently, affects the chemicals partitioning between water and plastic [76]. In the study performed by Velzeboer et al., an increase of salinity led to enhanced sorption of PCBs. Furthermore, the salinity may influence the aggregation state of the plastic particles, increasing this effect in the smallest ones. Therefore, the sorption of organic chemicals by MPs is expected to be different in freshwaters and marine environments. Xu et al. reported that the salinity did not affect the sorption of PDBEs when considering four types of MPs [81]. Similarly, Guo et al. concluded that the sorption capacity of sulfamethoxazole onto six plastic types (PET, PS, PP, PE, PA, and PVC) was reduced with a salinity increase. Overall, the salinity effect on sorption/desorption processes is dependent on the specific chemical-polymer interaction [99].

The presence of DOM could also affect the sorption processes of chemicals by plastics as DOM competes with other chemicals for adsorption sites on the surface of the plastics. For instance, Shen et al. observed that the humic acid pre-treatment decreased the tetracycline sorption by PE [100]. Furthermore, DOM enables changes in plastics properties due to their interaction, facilitating the interplay of plastics with hydrophilic chemicals. For instance, Zhang et al. concluded that the sorption of oxytetracycline on aged PS was promoted by the presence of humic acids. The authors exposed that the interaction of this polar pollutant via complexation could explain this fact [101]. The results observed by Qiao et al. also pointed out in the same vein. They noted that the presence of DOM promoted the adsorption of Cu on PS-MPs [102]. Despite that, other authors suggested that the presence of DOM may decrease the adsorption of antibiotics by PE [103].

These findings led to conclude that environmental influence on the sorption capacities of chemicals by plastics is strongly related to each specific interaction. For that reason, a better understanding of the sorption/desorption mechanisms between the most toxic chemicals (e.g., PCBs, PAHs, PBDEs) and the most abundant plastic types in marine environments (e.g., PE, PS, PP) is essential.

## 3. Effects of MPs-pollutants Interaction on Biota

**Figure 5:**
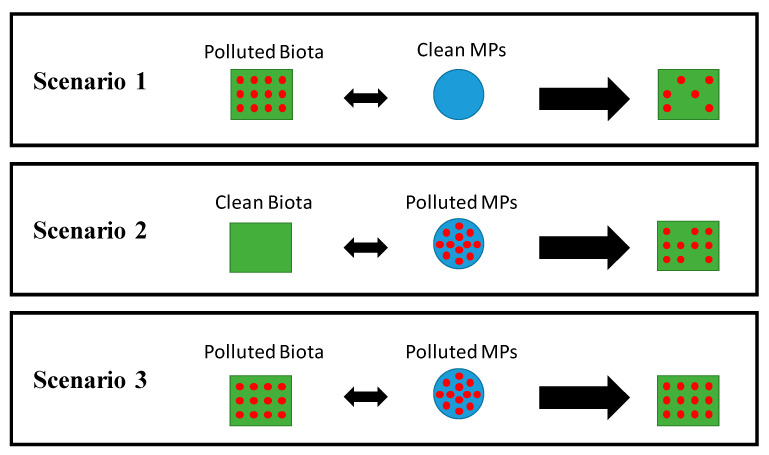
Schematic representation of the three possible bioaccumulation scenarios to assess the role of MPs (blue circles) as vectors of environmental pollutants (red dots) in marine biota (green squares).

As introduced above, MPs possess the ability to sorb organic chemicals due to their hydrophobic surface. Thereupon, pollutants may desorb from the plastics leading to an increment of their bioavailability to aquatic organisms. This capacity has led to the hypothesis that, in addition to direct effects on their interactions with biota (e.g., entanglement, ingestion), MPs might also play a significant role in aquatic ecotoxicology as vectors for toxic compounds. This hypothesis is still on the debate within the scientific literature. There are three possible scenarios which will be described below and graphically summarized in Figure 5. These different options should be analysed in-depth to accurately assess the real potential of MPs as carriers of hazardous chemicals and characterize the possible effects on the bioaccumulation and bioavailability of these compounds through MPs ingestion.

### 3.1. Scenario 1: Contaminated Biota Eats Clean Plastics

In this scenario, contaminated marine biota ingests clean microplastic pellets without sorbed environmental pollutants (see Figure 5). The ingestion of these clean microplastic beads may enable a chemical contaminant reduction in animals body. In other words, MPs could act as a sink for the bioaccumulation decrease. In this process, two simultaneous mechanisms may occur. Firstly, initial strong sorption of the chemicals to the MPs followed by desorption at a lower rate, allowing the decrease in the bioaccumulation of pollutants. Several authors have reported this cleaning effect. Koelmans et al. evaluated a conceptual model that simulated the impacts of plastic on the bioaccumulation of POPs. Results suggested increased bioaccumulation by ingestion of plastic-containing POPs and a decreased bioaccumulation by ingestion of clean plastics [64]. 

Similarly, Gouin et al. reported that bioaccumulation declined for compounds with a log K_ow_ between 6.5 and 7.5. The authors presented over a twenty per cent reduction in body burden concentrations as a consequence of including a ten per cent of MPs in the diet. These results can be justified by the high sorption affinity assumed by polyethene [104]. Granby et al. also described that for a wide variety of pollutants (e.g., PCBs, PBDEs, methyl mercury, PFOS, perfluorooctanoic acid (PFOA), PFOSA and perfluorononanoic acid (PFNA)), the presence of clean MPs in contaminated feed increased the elimination coefficient from European seabass (*Dicentrarchus labrax*) [105]. In contrast, Rummel et al. exposed that uncontaminated polyethylene microspheres had no significant effect on the depuration rates of PCBs in an in-vivo fish feeding experiment (rainbowfish, *Oncorhynchus mykiss*) [106].

Finally, it should be mentioned that this scenario is currently unlikely to occur. On the one hand, most of the manufactured plastics incorporate in their surface several chemicals to improve their properties. On the other hand, MPs that are ingested are likely to sorb a wide variety of environmental pollutants before being eaten by biota.

### 3.2. Scenario 2: Clean Biota Consumes Contaminated Plastics

This scenario is the opposite of the previous one in which non-contaminated marine animals ingest contaminated MPs. Most of the reported bioaccumulation studies consider this scenario because, in this case, the experimental exposures in the laboratory are easier to perform. For instance, Granby et al. reported that the presence of the MPs with sorbed pollutants (see details in the previous scenario) decreased their elimination coefficient in seabass compared to the feed containing only pollutants [105]. Avio et al. suggested that MPs could efficiently favour the bioaccumulation of pyrene in mussel tissues. In this work, the authors concluded that an elevated desorption and bioconcentration process of this chemical occurred from MPs to mussel under physiological gut conditions [107]. In addition, Teuten et al. also explained that feeding chicks of streaked shearwater (*Calonectris leucomelas*) with MPs naturally contaminated from the Tokyo Bay (PCBs concentrations ranging from 51 to 562 ng g^−1^, with a mean of 97 ng g^−1^) implied an enhancement of PCBs bioaccumulation [54]. Similarly, Rochman et al. reported that polyethylene ingestion enabled the increased bioaccumulation of PAHs, PCBs and PBDEs [31]. However, Devriese et al. concluded that three weeks of exposition to PCBs-loaded PE microspheres did not lead to significant bioaccumulation of these pollutants in the Norway lobster (*Nephrops norvegicus*) [108].

At this point, it should be highlighted that environmental pollutants such as POPs can be found almost everywhere. For this reason, recent studies have focused on the study of other emerging pollutants. For these chemicals, MPs could act as a vector to increase their bioavailability. For example, Zhang et al. suggested that polystyrene microspheres might enhance the bioaccumulation of roxithromycin, a semi-synthetic macrolide antibiotic, in the freshwater fish red tilapia (*Oreochromis niloticus*) [109]. Similarly, the concentrations found in loach liver tissue of the antidepressant venlafaxine and its metabolites were significantly higher for the coexposure treatment group (venlafaxine + PVC microplastic) in comparison to the exposure of the chemical alone [110]. Moreover, the metabolism of chemicals by metallothionein-like proteins (MTLP) was demonstrated to be inhibited by the presence of MPs [110]. In contrast, Beiras et al. reported that polyethylene MPs (with a nominal size between 4 and 6 µm) did not increase the bioaccumulation of nonylphenol (usually released in the environment by the degradation of the most common non-ionic surfactants used in detergents and cleaning products) and 4-Methylbenzylidene-camphor (4-MBC, which is widely used in UV filters and sunscreens) in marine zooplankton [111]. Another important group of chemicals in which this scenario may be relevant in bioaccumulation investigations are plastic additives, as MPs may well increase their bioavailability. Sala et al. assessed the organophosphorus flame-retardant (OPFR) levels in dolphins from Southern European waters. These compounds have been widely used as plastic additives since the 1960s. This study showed that OPFRs were found in the 100% of the marine mammals studied, and the total OPFRs levels reached up to 24.7 ng g^−1^. The authors concluded that marine plastic litter could be an important source of bioaccumulation and biomagnification of OPFRs substances into marine mammals which could explain the similar concentration levels in tissues to halogenated flame retardants despite the large differences in the production volume of these families of compounds (i.e., production volume of OPFRs used as flame-retardant is roughly the half of the halogenated flame retardants production volume). In addition, the authors pointed to the lower capacity of OPFRs to bioaccumulate and biomagnify, which could indicate other pollution sources complementary to their use as flame retardants [112]. Chen et al. investigated the Bisphenol-A (BPA) bioaccumulation enhancement in zebrafish due to the presence of NPs. Results demonstrated that the presence of NPs led to the highest levels of BPA in the head and viscera of the zebrafish. Conversely, BPA muscle and gill concentrations did not significantly vary among treatments [113].

These results lead to highlight the importance of analysing bioaccumulation rates in different tissues to better understand the hazard and risk posed by exposure to plastic particles in the presence of chemicals.

### 3.3. Scenario 3: Contaminated Biota Ingests Contaminated Plastics

Finally, the third scenario represents the most prevalent biota-MPs interaction as neither of the oceans and freshwaters systems in the world are clean enough [14,87,114]. For that reason, biota is likely to bioaccumulate organic pollutants such as POPs through respiration or their diet (i.e., biomagnification). MPs and marine biota presumably reached the sorption equilibrium before animals ingested them. Consequently, many authors argue that the ingestion of MPs located in the same ecosystem does not enhance the pollutants bioaccumulation on marine animals. For instance, Herzke et al. found that POPs concentration in liver and muscle tissues of northern fulmar (*Fulmarus glacialis*) did not differ between plastic ingestion subgroups. For that reason, the authors concluded that marine biota would tend to bioaccumulate POPs regardless of the MPs consumption [66]. 

Similarly, Khan et al. suggested that PE microbeads with a size range of 10–106 µm did not increase the Ag uptake and bioaccumulation in zebrafish (*Danio rerio*) adults [115]. Rochman et al. could not determine any relationship between several hydrophobic organic compounds (HOCs) sorbed in MPs (e.g., PCBs, BPA, and alkylphenols) and amphipods. In spite of these results, a positive correlation was established between plastic ingestion and bioaccumulation of 183–209 PBDEs congeners in fish tissues [116]. In this work, the authors also found that lower chlorinated PCB congeners (mono to tetra) were significantly higher in lantern fish in the gyre and positively correlated with plastic density. Their results demonstrated that MPs could be a source of exposure to lower chlorinated PCBs [117]. Tanaka et al. also concluded that MPs found in oceanic seabirds (short-tailed shearwaters, *Puffinus tenuirostris*) stomachs might be the cause of higher PDBEs bioaccumulation in their abdominal adipose tissue [118]. All these results might confirm the role of MPs as vectors for contaminants in aquatic ecosystems, reflecting the main conclusion reported in Section 2. Therefore, as sorption/desorption mechanisms strongly depend on the specific plastic-chemical interactions, in addition of the particular environmental conditions, it is possible that contradictory results can be seen when several field investigations are compared due to the inherent complexity of the studies performed.

Summarizing, the influence of MPs on the bioavailability of environmental pollutants should not be underestimated, as it has been demonstrated that their effect is not negligible. The complexity of these studies caused by several factors that may influence the sorption/desorption mechanisms produce that contradictory results are obtained in similar bioaccumulation investigations. Therefore, these different scenarios should be deeply analysed to extract more accurate information about the real ecotoxicological influence of the MPs “Trojan horse” effect. It is also important to keep in mind that the presence of MPs may also enhance the bioavailability of emerging pollutants (e.g., pharmaceuticals, flame-retardant compounds, and plasticizers). For this reason, the lack of bioaccumulation and biomagnification research of these compounds due to MPs desorption should be urgently redressed.

## 4. Ecotoxicological Effects of MPs Combined with Sorbed Chemicals on Biota

Many recent publications have also focused on the assessment of the ecotoxicological effects that the pollutant-MPs interaction could cause on marine biota in comparison with the individual impacts produced for both contaminants. In other words, the potential synergistic or antagonistic effect that their interaction can produce is nowadays a new subject of study.

In Table 1, studies dealing with the interaction between MPs and inorganic pollutants are presented. Most of these works evaluated the acute toxicity of the individual pollutants and their mixture by evaluating toxicological parameters such as the survival rate, grown inhibition and post-predatory performance. Otherwise, only a reduced number of studies analysed chronic toxicity of the metal-MPs interaction. In these works, the most common assay is the assessment of several known biomarkers involved in crucial functions for survival as oxidative stress, neurotoxicity or immune responses (e.g., acetylcholinesterase (AChE), superoxide dismutase (SOD), lipid peroxidation (LPO), 7-ethoxyresorufin O-deethylase (EROD)). Almost all the research exposed that mixtures MPs-pollutant enhanced the toxicity in comparison to the individual pollutants. Despite this fact, some studies do not lead to that conclusion. Davarpanah et al. found that in the range of the concentrations analysed (0.02 to 0.64 mg Cu/L), the toxicity of copper did not increase when it was combined with MPs (0.184 mg/L) [119]. Fu et al. also studied the MPs-Cu interaction (10 mg MPs/L combined with 0.5 mg Cu/L) on microalgae. In contrast to Davarpanah, their results concluded that several toxicity parameters suffered a significant increase after ten days of exposure [92]. These conflicting conclusions could be explained due to the different range of studied MPs concentrations as well as the exposure time. Consequently, as some of the tested concentration levels were environmentally relevant, the considered exposure time may be insufficient to obtain significant changes in the average specific growth rate of *Tetraselmis chuii.*

POPs are by far the group of chemicals most studied when considering the toxicity of their interaction with MPs. Several families of compounds can be highlighted such as PAHs (e.g., phenanthrene, fluoranthene), PCBs, PBDEs and polyfluoroalkyl substances (PFAS). Table 2 shows that most of these studies concluded that the mixture of both pollutants caused higher toxic effects in comparison to the individual exposition. However, other studies, such as the one presented by Guven et al., did not reach the same conclusions as to the combined exposure of pyrene and PS-divinylbenzene plastic microspheres did not magnify the PAH acute single impact [126]. Again, differences between studies can be attributed to several factors such as the different exposure times, concentration levels used for both pollutants, and analysed organisms.

It is important to highlight that the chronic exposure evaluation of POPs–MPs toxic interaction was more studied compared to other groups of chemicals such as heavy metals or pharmaceuticals. Moreover, there are some studies in which their exposure mixtures were prepared in the real environment [127]. Other relevant differences between these studies are the evaluated toxicological parameters. Here, gene expression analysis and biomarker performance changes are the most common. In a few cases, the survival rate or the post-predatory assay were considered.

Finally, the synergistic or antagonistic effects of the combination of MPs with other organic chemicals produced in biota are summarized in Table 3. Organic pesticides (e.g., chlorpyrifos), plasticizers (e.g., BPA) or pharmaceuticals (e.g., roxithromycin) are examples of these alternative chemical families. Similarly to heavy metals-MPs studies, most of these works considered an acute toxicity evaluation of the interaction. Hence, mortality rates, grown inhibition and morphology changes are the most studied toxicological parameters. As shown in Table 3, toxicological results lead to the conclusion that MPs enhance the ecotoxic effects of the chemicals tested.

The overall results led to conclude that the interaction between MPs and the different groups of environmental pollutants cannot be neglected under standard experimental conditions. However, it is still difficult to achieve a conclusion regarding if these results are likely to mimic environmental conditions. This concern occurs as most of the experimental studies use concentration levels that are not always consistent with the concentrations found in environmental conditions, as they are usually higher. Moreover, several microplastic toxic analyses include the use of an ultrasonic bath or surfactants (e.g., Tween-20 or Tween-80) to obtain the dispersion of the MPs and maintain homogeneous suspensions. For that reason, observed toxic effects in aquatic organisms may correspond to high pollutant stress. Despite that, as highlighted in the work by Ngoc et al., MPs and NPs concentration levels in marine environments are not likely to decrease. For that reason, the study of the biological impacts on organisms after acute exposition may also be of great interest [128]. Ideally, a realistic scenario, in which several types of MPs are exposed to different mixtures of chemicals in environmentally relevant concentrations should always be performed. Moreover, testing several organisms which represent different levels of the food chain (e.g., zooplankton, microalgae, early juveniles and fish) is also required. Besides, the toxicological assessment on cell cultures could provide in vitro models to model the effects of these environmental pollutants at the cellular level. Unfortunately, these types of studies cannot be easily performed. Many reasons prevent these studies, such as the complexity of the experimental work or the lack of animal models.

Most toxicological works use PE and PS (with fewer examples employing PVC) to conduct the individual and interaction studies. This selection is consistent with the polymeric amount found in the marine plastic debris where both PE and PS are the most detected polymers. Additionally, the study of both individual and combined effects of pollutants with bioplastics may be beneficial as their production is currently increasing. The size-dependent effect should also be urgently assessed. As several toxicological studies reported [61,113,129], smaller plastic particles cause enhanced harmful effects. For this reason, the assessment of the toxicological impact of NPs and their interaction with other environmental pollutants is also needed. Similar studies to those performed by Kim [120] and Fu [92] should be carried out, providing an in-depth evaluation of the combined effects of pollutants with ageing plastics. Due to the high persistence of these compounds, the belief that most of the plastic debris present in marine ecosystems have been exposed to several degradation processes seems logical.

Considering the variety of studied chemicals, PAHs, PCBs and metals interaction with MPs and NPs are the most usual studies. Besides, the combined toxicity of MPs with emerging pollutants (mainly PhACs) has increased in recent years. However, it is important to remark that the assessment of the synergetic or antagonistic toxic effects of environmental pollutants combined with MPs using a mixture of chemicals may be the key to understand the actual toxicity of MPs. The sorption enhancement or decline of chemicals in the MPs matrix in addition to the different interaction mechanisms with biota would provide more realistic conclusions. Thus, the approaches performed by Rochman et al. [31,127], Granby et al. [105] or Rainieri et al. [136] should be adopted where possible in future researches.

It is also essential to keep in mind that there are different ways to evaluate the combined effects of chemical pollutants and MPs. On the one hand, the majority of works have studied the combined toxicological impact by performing a co-exposure of both pollutants. On the other hand, other studies have tested the combined toxicity carrying out a previous incubation step. Consequently, these studies also allow the assessment of the possible desorption of pollutants from the MPs. For this reason, this kind of studies could be especially important for those chemicals commonly related to plastic compounds. For instance, the combined effects of MPs with plasticizers such as BPA, TBBPA or dibutyl phthalate should also be performed by adding a previous incubation step. Therefore, these toxicological studies would model better the real scenarios as those compounds could also be leached from the MPs matrix and surface.

Regarding the analysed toxicological parameters, most of them are related to the evaluation of acute toxicity. For instance, survival rate, post-predatory performance or grown inhibition are the most used. In addition, biomarkers evaluation (e.g., changes of AChE, glutathione (GSH), SOD, malondialdehyde (MDA)) is also widely performed. However, future studies should also integrate the information coming from different omic technologies (e.g., transcriptomics, metabolomics) to understand the changes caused by the interaction of MPs and pollutants at a molecular level. Besides, the use of these multi-omics approaches may also be of great help to achieve a holistic view of the whole biological picture. These multi-omics studies are suitable for the confirmation of the causal relationship and association between environmental exposure and pathogenesis, by assessing multiple aspects following the omics cascade: mutations in the DNA sequence may induce changes in the degree of epigenetic regulation, which might alter gene expression and, consequently, protein expression, leading eventually to a metabolomics effect [150].

## Figures and Tables

**Table 1:** Studies assessing the synergetic/antagonistic effect of inorganic pollutants combined with MPs.

MP Type	MP Size	Chemical Sorbate	Exposure Concentrations *	Exposure Time	Organism	Toxicological Assessment	Highlight Results	Ref.
PS	Average diameter: 201.5 nm	Nickel	Ni alone [Ni] = 1, 2, 3, 4, and 5 mg/L MPs alone [MPs] = 1,5,10,20,30 mg/L Variable Ni-Fixed MPs [Ni] = 1, 2, 3, 4, and 5 mg/L [MPs]= 5 mg/L Fixed Ni-Variable MPs [Ni] = 3 mg/L [MPs] = 1,5,10,20,30 mg/L Variable Ni-Variable MPs 1 mg/L Ni-1 mg/L MP 2 mg/L Ni-5 mg/ L MP 5 mg/L Ni-30 mg/L MP	48 h	*Daphnia magna*	Rate of abnormalities and changes in the morphology Rate of immobilization	Enhanced toxicity of Ni in combination with both MPs Higher immobilization effect for Ni - PS-COOH exposure Ni showed an antagonistic effect on toxicity with PS and synergistic with PS-COOH Results may indicate that the toxic effects of MPs and Ni vary depending of the properties of both pollutants	[120]
PS-COOH	Average diameter: 191.3 nm							
PE	1–5 µm	Chromium (VI)	Cr (VI) alone [Cr (VI)] = 0, 5.6, 8.4, 12.6, 18.9 and 28.4 mg/L MPs alone [MPs]= 0.184 mg/L MPs + Cr (VI) Co-exposure performed using the same concentrations of single treatments	96 h	Early juveniles of the common goby fish (*Pomatoschistus microps*)	Post-predatory performance assay The activities of AChE, GST, EROD activities LPO levels	Significant decrease of the predatory performance and significant inhibition of AChE activity under simultaneous exposure Long-term exposure to different environmental conditions in developmental phases influences the response of early juveniles	[121]
PS	32–40 µm	Cadmium	Cd alone [Cd]= 0 and 50 µg/L MPs alone [MPs]= 0, 50, 500 µg/L MPs + Cd 3 × 2 array configuration (MPs previously preloaded with Cd for 24 h before the exposure experiments)	30 days	Early juveniles of discus fish (*Symphysodon aequifasciatus*)	Survival rate Body length The activities of SOD, CAT, GPx, LZM, ACP and ALP The level of GSH and C3 The concentrations of MDA and PC	The MP + Cd mixture induced severe oxidative damage as well as the stimulation of the immune system Co-exposure stimulate the innate immune responses of early juveniles	[122]
PS	5 µm	Cadmium	Cd alone [Cd]= 10 µg/L MPs + Cd 10 µg/L Cd-20 µg/L MPs 10 µg/L Cd-200 µg/L MPs (MPs incubated during 96h before the exposure experiments)	3 weeks	Zebrafish (*Danio rerio*)	Histological analysis (liver, gut and gills) GSH and MT levels SOD activity mRNA levels of 8 target genes in zebrafish tissues	Enhanced toxicity of Cd in combination with MPs Oxidative stress and early inflammatory responses observed in the mixture treatments Important changes in the gene expression observed for all co-exposure treatments	[123]
unknown	1–5 µm	Mercury	Hg alone [Hg]= 0.010 and 0.016 mg/L MPs alone [MPs]= 0.26 and 0.69 mg/L MPs + Hg 4 binary mixtures using the same concentrations of single exposures	96 h	Juvenile European seabass (*Dicentrarchus labrax*)	AChE, ChE, IDH and LDH activities LPO levels	A significant interaction between MPs and Hg was achieved Biomarkers’ variation was highly influenced by the concentration of MPs	[124]
unknown	1–5 µm	Mercury	Hg alone [Hg] = 30 µg/L MPs alone [MPs] = 0.13 mg/L MPs + Hg Co-exposure performed using the same concentrations of single treatments	8 days (+ 6 days in clean medium)	Freshwater bivalve (*Corbicula fluminea*)	The post-exposure filtration rate ChE, IDH, GST, GSR, GPx, ODH and CAT activities LPO levels	Antagonistic behaviour between MPs and Hg in several biomarkers Six days of post-exposure recovery in the clean medium was not enough to reverse the toxic effects induced by both pollutants	[125]
PE	10–45 µm	Mercury	Hg alone [Hg] = 10 µg/L MPs alone [MPs] = 25 µg/L MPs + Hg Co-exposure and incubation treatments performed using the same concentrations of single treatments (incubation for 96h)	7 days	Manila clam (*Ruditapes philippinarum*)	Histological analysis (gill and digestive gland) Filtration rates Immunomodulation Oxidative stress	The filtration rates decreased as a result of the co-exposure A higher decrease in haemocyte viability was detected in co-exposure treatments Antioxidant parameters remain unchanged in the mixture in comparison to single treatments	[7]
PE	1–5 µm	Copper	Cu alone [Cu]= 0.02, 0.04, 0.08, 0.16, 0.32 and 0.64 mg/L MPs alone [MPs] = 0.046, 0.092, 0.184, 0.368, 0.736 and 1.472 mg/L MPs + Cu 6 binary mixtures using the same concentrations of Cu combined with 0.184 mg/L of MPs	96 h	Marine microalgae (*Tetraselmis chuii*)	The average specific growth rate and the percentage of growth inhibition	No significant differences were observed between treatments with and without MPs MPs did not influence the Cu toxicity	[119]
Virgin PVC	D50: 139 µm	Copper	Cu alone [Cu]= 0, 0.2, 0.5 and 1 mg/L MPs alone (virgin and aged) [MPs] = 10, 100 and 1000 mg/L MPs + Cu 0.5 mg/L Cu-10 mg/L aged MPs	10 days	Microalgae (*Chlorella vulgaris*)	The growth inhibition ratio (IR) and biomass productivity The enzymatic activities of SOD and MDA	Mixture exposure enhances the cell growth in comparison to single treatments The ageing of MPs poses stronger inhibitory effects in microalgae than virgin pellets	[92]
Aged PVC	D50:132 µm							
PS	0.1 µm 20 µm	Copper	Cu alone [Cu] = 50 µg/L MPs alone [MPs] = 200 µg/L MPs + Cu Combination of concentrations used in single treatments	14 days	Zebrafish (*Danio rerio*)	SOD, MDA and MT levels Transcriptomic analysis	Synergetic effects in co-exposure treatments of small MPs were observed The presence of MPs and DOM aggravates the Cu-toxicity	[102]

* Shadowed cells represent environmental relevant concentrations.

**Table 2:** Studies assessing the possible synergetic/antagonistic effect of persistent organic compounds (POPs) combined with MPs.

MP Type	MP Size	Chemical Sorbate	Exposure Concentrations	Exposure Time	Organism	Toxicological Assessment	Highlight Results	Ref.
PS-divinilbenzene	97 μm	Pyrene	Pyrene alone [Pyrene] = 0.1 µM MPs alone [MPs] = 100 particles/L MPs + Pyrene 100 nM pyrene + 100 particles/L MPs	24 h	Tropical fish juveniles (*Lates calcarifer*)	Mortality rate Juveniles behaviour Predatory performance Size differences	Individuals exposed to both pollutants were the most affected group, but the negative impact was relatively small	[126]
PE	< 100 µm	Pyrene	MPs alone [MPs]= 20 g/L MPs + pyrene Before the experiment, a solution of PE or PS were incubated with pyrene (50 µg/L) for 6 days	7 days	Marine mussel (Mytilus *galloprovincialis*)	Histological analysis (gills and digestive glands) Gene transcription analyses Genotoxic effects Immunological alterations Neurotoxic responses Oxidative stress Antioxidant defences	Clear separation between control and MPs exposed mussels Biological variations were influenced by the typology of polymer (PE vs PS) Only genotoxic responses separated virgin from pyrene- contaminated polymers	[107]
PS								
PE	1–5 µm	Pyrene	Pyrene alone [Pyrene]= 20 and 200 µg/L MPs alone [MPs]= 0, 18.4 and 184 µg/L MPs + Pyrene 0 µg/L pyrene-18.4 µg/L MPs 200 µg/L pyrene-184 µg/L MPs 200 µg/L pyrene-184 µg/L MPs	96 h	Juveniles of the common Goby (*Pomatoschitus microps*)	Protein content AChE, IDH, GST activities - LPO levels Bile samples were analysed for pyrene metabolites	The presence of MPs was found to delay the pyrene-induced mortality Enhanced concentration of pyrene-metabolites was detected in co-exposure treatments Results suggest toxicologically relevant interactions between both pollutants	[130]
PE	10–90 µm	Fluoranthene (Flu.)	Flu. alone [Flu]= 100 µg/L MPs alone [MPs]= 1000 particles/mL MPs + Flu. Flu – PE/PHB co-exposure or incubation at the same concentrations tested in single exposures (incubation during overnight)	96 h	Blue Mussel (*Mytilus edulis*)	Protein content in the cytosol The cytosolic concentration of GSH SOD, CAT, GPx and SeGPx activities	In co-exposure and incubation treatments, biochemical responses were generally comparable with those exerted MPs only Apparent absence of combined effects of MPs with the pollutant.	[5]
PHB	10–90 µm							
PE	10–90 µm	Fluoranthene (Flu.)	Flu. alone [Flu]= 50 and 100 µg/L MPs alone [MPs]= 100 and 1000 particles/mL MPs+ Flu. 50 µg/L Flu.-100 particle/mL 100 µg/L Flu.-1000 particle/mL (For both mixtures, co-exposure and incubation experiments (incubation during overnight))	96 h	Blue mussel (*Mytilus edulis*)	Total GSH + 2GSSG levels SOD, CAT, GPx and SeGPx activities	No synergistic or antagonistic effect was seen in the co-exposure or the incubation experiments	[131]
PS	Mix of 2 and 6 µm	Fluoranthene (Flu.)	Flu. alone [Flu]= 30 µg/L day MPs alone [MPs]= 32 mg/L day MPs + Flu. 30 µg/L day Flu.-32 mg/L day PS	7 days (+ 7 days of depuration)	Marine mussel (*Mytilus spp.*)	Morphological and functional analyses of hemocytes Hemocyte mortality Circulating hemocytes concentration Phagocytosis activity Histopathological assessment (digestive tract and intestine) ROS production Levels of LPO SOD, CAT, GR and GST activities Gene expression analysis	Increase in the total histopathological lesions/ abnormalities was demonstrated in co-exposure treatments After depuration, a higher fluoranthene concentration was detected in mussels exposed to the mixture of MPs and Flu Results suggested that MPs led to modulated fluoranthene kinetics and toxicity in marine mussels.	[132]
PS	500 nm 30 µm	Benzo[a]pyrene (B[a]P) 17β-estradiol (E2)	B[a]P alone [B[a] P] = 5 and 50 mg/L E2 alone [E2] = 0.1 and 1 mg/L MPs alone [MPs]= 1 mg/L MPs + Pollutant Combination of individual concentrations of MPs of both sizes and the organic contaminants	4 days	Bivalve specie (*Tegillarca granosa*)	Analysis of total counts, cell-type composition, and phagocytic activity of haemocytes ROS and Ca2+ concentration from haemocytes LZM content and activity Gene expression of three major types of genes	POPs toxicity was aggravated by smaller MPs and mitigated by larger MPs The deleterious impacts of B[a]P or E2 were mitigated by the presence of larger sized MPs and aggravated smaller ones	[8]
LD-PE	20–25 µm	Benzo(a)pyrene (B[a]P)	B[a]P alone [B[a] P] = 150 µg/L MPs alone [MPs]= 10 mg/L MPs+ B[a]P 15 µg/g B[a]P-10 mg/L MPs (To reach this B[a]P sorbed concentration, 2 days of incubation was performed)	7, 14 and 28 days	Marine mussel (*Mytilus galloprovincialis*)	Immunological alterations of hemocytes Neurotoxic responses in hemocytes and gills Oxidative stress Antioxidant defences, Genotoxic effects Transcriptional responses	The overall evaluation provided a clear separation between times and typologies of exposure Significant alterations measured on the immune system Results suggested that the toxicological risk of MPs for marine organisms is probably low, but not negligible	[133]
PE	212–250 µm	Phenanthrene (Phe.) Anthracene	Phe. alone [Phe.] = 0.12 µM Anthracene alone [Anthracene] = 0.14 µM MPs alone [MPs]= 0.02 and 0.2 g/g sediment MPs + Phe. / Anthracene Lower dose of PE combined with pollutants preloaded for 96h	2 weeks	Bacterial community of sediments	Gene expression assessment	The presence of MP reduced the effect of the two PAHs on microbial community composition and the degradation of these organic compounds	[134]
LD-PE non-uniformly shaped	< 60 µm	Phenanthrene (Phe.)	Phe. alone [Phe.] = 10 and 100 µg/L MPs alone [MPs]= 50 and 500 µg/L MPs + Phe. Combination of individual concentrations of MPs the organic contaminant	96 h	African catfish (*Clarias gariepinus*)	Histopathological analysis (liver and gill) Glycogen stores of the liver Biomarkers responses of AST, ALT, LDH, ALP, γGT Contents of total protein, total albumin, lipase, glucose, lactate, direct bilirubin, HDL, LDL, TG and cholesterol Gene expression analysis	Changes in biomarker responses of co-exposure treatment might be due to the facilitated transportation of Phe into the fish body Findings suggested toxicologically relevant interactions between MPs and Phe	[135]
PE	50 nm 500nm 5 µm 10 µm 15 µm	Phenanthrene (Phe.)	Phe. alone [Phe.] = 0, 0.05, 0.1, 0.2, 0.4, 0.8 and 1.2 mg/L MPs alone [MPs]= 0, 2.5, 5, 10 and 50 mg/L NPs alone [NPs]= 0, 2.5, 5, 8.5, 11 and 14.5 mg/L MPs/NPs + Phe. Combination of the individual concentrations tested for both pollutants	48 h	*Daphnia magna*	Immobilization rate of the daphnids	Enhanced immobilization of daphnia was observed in co-exposure treatments (especially for NPs) The presence of NPs inhibited the dissipation of phenanthrene of the environment	[61]
LD-PE	125–250 µm	α-HBCD 2,4,6-tribromophenol PBDEs mix (PBDE 47, 99, 153, 154) PCB congeners (28, 52, 101, 118, 138, 153, 180) methyl mercury PFOS PFOA PFOSA PFNA	Feed A: Basic feed (control) Feed B: Basic feed + contaminants sorbed to MPs before the incorporation of 2% into pellets (incubation for overnight) Feed C: Basic feed + contaminants without MPs Feed D: Basic feed + contaminants and clean MPs	80 days (+ 51 days of depuration)	European seabass (*Dicentrarchus labrax*)	Growth factors Feeding rates Gene expression analysis	Results indicated that MPs inhibit or induce detoxification in the liver and influence the lipid distribution Gene expression results also indicated that MPs might indeed potentiate the adverse effect of some chemical contaminants	[105]
LD-PE	125–250 µm	Methylmercury Perfluoroctanesulfonate Perfluorooctanoat PFOSA PFNA α-HBCD 2,4,6-Tribromphenol PBDE 47 PBDE 99 PBDE 153 PBDE 154 PCB 28 PCB 52 PCB 101 PCB 118 PCB 138 PCB 153 PCB 180	Feed A: Basic feed Feed B: Basic feed + 4% of clean MPs Feed C: Basic feed + 2% of MPs with sorbed POPs Feed D: Basic feed + POPs	3 weeks	Zebrafish (*Danio rerio*)	Visual observation (microscopic level and Hispathological analysis) Evaluation of differential gene expression of some selected biomarkers	Feed C produced the most evident effects, especially on the liver Combined effects of MPs and chemicals significantly altered the homeostasis in greater manner respect both pollutants alone	[136]
LD-PE	marine exposition: 3 mm feed exposition: 0.5 mm	PAHs, PCBs and PBDEs congeners	Feed A: Basic feed Feed B: Basic feed + virgin LD-PE Feed C: Basic feed + marine-plastic treatment (LDPE deployed in San Diego Bay for 3 months)	2 months	Japanese medaka (*Oryzias latipes*)	Histopathological analysis (gonads) Gene expression analysis on selected liver’s genes and biomarkers for endocrine disruption	Results show early- warning signs of endocrine disruption in fish exposed to a mixture of plastic and sorbed contaminants	[127]
LD-PE	marine exposition: 3 mm feed exposition: 0.5 mm	PAHs, PCBs and PBDEs congeners	Feed A: Basic feed Feed B: Basic feed + virgin LD-PE Feed C: Basic feed + marine-plastic treatment (LDPE deployed in San Diego Bay for 3 months)	2 months	Japanese medaka (*Oryzias latipes*)	Histopathological analysis (gonads) Gene expression analysis on selected liver’s genes and biomarkers for endocrine disruption	Hepatic stress in medaka exposed to the combination of plastic and sorbed contaminants was demonstrated No significant differences in the expression of CYP1A were found between treatments	[31]

* Shadowed cells represent environmental relevant concentrations.

**Table 3:** Studies assessing the possible synergetic/antagonistic effect of different types of emerging pollutants combined with MPs.

MP Type	MP Size	Chemical Sorbate	Exposure Concentrations	Exposure Time	Organism	Toxicological Assessment	Highlight Results	Ref.
**Pesticides**								
HD-PE with irregular shape	mean size: 7.73 µm	Chlorpyrifos (CPF)	CPF alone [CPF] = 0, 0.1, 1, 10, 100 µg/L MPs alone [MPs]= 0, 0.1, 1, 10, 100 µg/L MPs + CPF 100 µg/L CPF-100 µg/L MPs co-exposure and incubation treatments (incubated for 2 h)	48 h	Marine copepod (*Acartia tonsa*)	The survival rates Fecundity, feeding and egg viability	CPF showed higher toxicity when combined with MP than alone for all tested biological responses Higher toxicity was observed with the co-exposure treatment	[137]
PE	mean size: ranging from 2–6 µm maximum particle size: 22 µm	Chlorpyrifos	CPF alone [CPF] = 0 to 4 mg/L MPs alone [MPs]= 0.5, 1, 10 and 25 mg/L MPs + CPF (co-exposure) 0–3 mg/L CPF-1 mg/L MPs co-exposure and incubation treatments (incubated for 2 h)	72 h	Microalgae (*Isochrysis galbana* clone T-ISO)	Microalgae daily growth rate Inhibition of microalgae growth	MPs reduced the toxicity of CPF MPs were not small enough to penetrate the microalgal cell and cause any damage	[138]
PS	0.1 mm, 0.55 mm 5 mm	Triphenyltin chloride (TPTCl)	TPTCl alone [TPTCl] = 30 μg/L MPs alone [MPs]= 0.05, 0.5, 5 mg/L MPs + TPTCl Combination of the individual concentrations tested for both pollutants	96 h	Microalgae (*Chlorella pyrenoidosa*)	Morphology and structural damage Grown inhibition	PS particles toxicity to the green algae was size-dependent Toxicity of the mixture was size-dependent: MPs with smaller particle size increased the toxicity of TPTCl	[139]
Pristine PE	10–27 µm	Bifenthrin	Bifenthrin alone [Bifenthrin] = 0.1 to 3.2 µg/L MPs alone [MPs]= 5 mg/L MPs+ Bifentrin (co-exposure) 0.1–3.2 µg/L CPF-5 mg/L MPs	48 h	Freshwater larvae organism *(Chironomus tepperi*)	Immobilization rates	The addition of MPs to synthetic water reduced the toxicity of bifenthrin The addition of MPs to river water did not mitigate bifenthrin toxicity due to the greater interaction of bifenthrin with DOM	[140]
PET/PA fibers	length: 10 µm width: 2 µm	Three different glyphosate chemical formulations	Glyphosate alone [Glyphosate] = 2.5 mg/L PE alone [MPs]= 0.01 mg/mL Fibers alone [MPs]= 0.045–0.136 µg/L MPs + glyphosate Single treatments were combined	1 week	*Daphnia magna*	Mortality rate	The toxicity of the mixture was more influenced by the type and size of the MPs than their abundance Toxicity of glyphosate was enhanced by the presence of MPs	[141]
PE	1–10 µm							
PS	1 µm	Dimetholate Deltamethrin	Pesticides alone [Dimetholate] = 0.156,0.313, 0.625, 1.25 and 5 mg/L [Deltamethrin]: 0.016, 0.08, 0.4, 2,5,10 µg/L MPs alone [MPs]= 300,000 particles/mL MPs + glyphosate Single treatments were combined	72 h	*Daphnia magna*	Mortality rate Impaired mobility	The concentrations at which detrimental effects occurred were not influenced by the presence of MPs	[142]
**Pharmaceuticals**								
PE	1–5 µm	Cefalexin	Cefalexin alone [Cefalexin]= 1.3, 2.5, 5 and 10 mg/L MPs alone [MPs]= 0.184 mg/L MPs + Cefalexin Combination of individual exposure concentrations (Exposure experiments performed at 20 and 25 °C)	96 h	Common goby juveniles (*Pomatoschistus microps*)	Mortality rate Post-predatory performance AChE activity LPO levels	The temperature rise increased the toxicity for both pollutants alone and in MPs mix No significant differences between cefalexin treatment alone and in MPs mix	[143]
PE	10–90 µm	Triclosan	Triclosan alone [Triclosan] = 0–300 µg/L MPs alone [MPs]= 0–25,000 MPs/mL MPs+ Cefalexin Combination of individual exposure concentrations of Triclosan and 500 MPs/mL	48 h	Marine copepod (*Acartia tonsa*)	Mortality of marine copepods	The LC50-values of individual pollutants and mixture were significantly different (synergistic effect)	[144]
PS	1 µm 10 µm	Roxithromycin (ROX)	ROX alone [ROX] = 0.1, 1, 5, 10, 50, 100, and 150 mg/L MPs alone [MPs] = 0.005, 0.05, 0.1, 0.2, 2, 15, 20, 25, 30, 35, and 40 mg/L MPs + ROX Mix 1: 0.1 mg/L 1-μm PS + 0.01 mg/L ROX Mix 2: 0.1 mg/L 10-μm PS + 0.01 mg/L ROX.	48 h	*Daphnia magna*	Mortality rate MDA levels Activities of: SOD, CAT, GST and GPx	Small-size PS was more toxic to D. magna than the large-size PS Co-exposure to 1-μm PS and ROX led to the strongest biological responses in D. magna	[6]
PS	0.1 µm	Roxithromycin (ROX)	Roxithromycin alone [ROX] = 50 µg/L MPs + ROX Mix 1: 1 µg/L MPs+50 µg/L ROX Mix 2: 10 µg/L MPs + 50 µg/L ROX. Mix 3: 100 µg/L MPs + 50 µg/L ROX.	14 days	Water fish red tilapia (*Oreochromis niloticus*)	Histopathological analysis (liver, gills, guts and brain) AChE, EROD, BFCOD, SOD and MDA activities	The neurotoxicity caused by ROX was alleviated due to the presence of MPs The presence of MPs may affect the metabolism of ROX in tilapia Oxidative damage in situations of co-exposure to MPs and ROX was mitigated in fish livers This study suggests that the effects of MPs combined with other pollutants cannot be ignored	[109]
unknown	1–5 µm	Florfenicol	Florfenicol alone [Florfenicol] = 1.8 and 7.1 mg/L MPs alone [MPs] = 0.2 and 0.7 mg/L MPs + Florfenicol Combination of individual exposure concentrations of both pollutants	96 h	Marine bivalve (*Corbicula fluminea*)	Feeding inhibition Histopathological alterations (digestive system and gills) Enzymatic activities of ChE, IDH, ODH, GST, GR, GPx and CATLPO levels	Enhanced toxicity of florfenicol in combination with MPs Differences in the toxicological effects induced by mixtures containing the lowest or the highest concentrations of both substances	[48]
PVC	< 10 µm	Venlafaxine O-desmethylvenlafaxine	Venlafaxine and derivate alone [Venlafaxine] = 0–500 µg/L O-desmethylvenlafaxine alone [O-desmethylvenlafaxine] = 0–500 µg/L MPs + chemicals Combination of individual exposure concentrations of both pollutants and 50 mg/L of MPs	4 days	Loach *(Misgurnus anguillicaudatus)*	SOD and MDA activities	In liver subcellular structure, MPs may help to transport pollutants into subtle areas and postpone the contaminants metabolism Mixtures enhance the oxidative stress in loach Enantioselective effects were observed in high dose exposure groups MPs combined with chemicals might cause more adverse effects to organisms compared with only chemicals themselves.	[110]
unknown	1–5 µm	Procainamide Doxycycline	Procainamide alone [Procainamide] = 4, 8, 16, 32, 64, 128 and 256 mg/l Doxycycline alone [Doxicycline] = 4, 8, 16, 32, 64 and 128 mg/l MPs alone [MPs] = 0.75, 1.5, 3, 6, 12, 24 and 48 mg/l MPs + chemicals Combination of individual exposure concentrations of both chemicals and 1.5 mg/L of MPs	96 h	Marine microalga (*Tetraselmis chuii*)	Inhibition of average specific grow per day Chlorophyll concentration decrease	Significant toxicity enhancement of each pharmaceutical in mixture with MPs was found for procainamide (chlorophyll), and doxycycline (both parameters)	[145]
PS	30 µm 500 nm	Sertraline (Ser)	Ser. alone [Ser]=100ng/L MPs alone [MPs]= 0.29 mg/L MPs+ Ser. Combination of individual exposure concentrations of both pollutants	14 days	Bivalve mollusk (*Tegillarca granosa*)	ROS generation Apoptosis status MDA, ACh and GABA levels Plama cortisol content ATP content and PK activity Transcriptomic analysis	Evident synergistic immuno-toxic effect was observed between Ser. and NPs NPs could exert more toxic effects than larger MPs	[146]
**Others (UV Filters, Surfactants, Plasticizers, …)**								
PE irregular shape	3.4 µm 9.9 µm	4-Nonylphenol (4-NP) 4- MBC	4-NP alone [4-NP] = 4, 25 and 70 µg/l 4-Nonyphenol alone [4-MBC] = 70, 150 and 350 µg/l MPs + chemicals Combination of individual exposure concentrations of both chemicals with 1 and 10 mg/L of MPs	48 h	Marine zooplanktons	Effective concentration reducing the larval size Mortality rate	The presence of MPs did not increase the toxicity of both chemicals tested	[111]
PE irregular shape	3.4 µm 9.9 µm	4-Nonylphenol (4-NP)	4-NP alone [4-NP] = 20 and 60 µg/l MPs+ 4-NP Combination of individual exposure concentrations of 4-NP with 1 and 10 mg/L of MPs	48 h	Planktonic sea-urchin larvae	Filtering rate Effective concentration reducing larval growth	The ingestion of MPs did not increase the toxicity of 4-NP	[147]
PE	50 nm	BPA	BPA alone [BPA] = 0.78 and 1 µg/l NPs alone [NPs]= 1 mg/l NPs+ BPA 1 µg/L BPA + 1 mg/L NPs	3 days	Zebrafish (*Danio rerio*)	Gene expression analysis AChE activity Dopamine level Protein content	The co-exposure of NPs and BPA led to increased neurotoxic effects in both CNS and dopaminergic system The reduction of the AChE activity in co-exposure treatment was alleviated in comparison to single experiments	[113]
PS	0.1 mm 0.55 mm 5 mm	Dibutyl phthalate (DBP)	DBP alone [DBP] = 0.25, 0.5, 1, 2, 4, 8 and 16 mg/l MPs alone [MPs]= 0.5, 1, 2, 4, 8, 16, 32 and 64 mg/l MPs + BPA Combination of individual exposure concentrations of both pollutants (MPs size: 0.1 mm)	96 h	Microalgae (*Chlorella pyrenoidosa*)	Grow inhibition rate Changes in morphology and structural damage Chlorophyll levels	The interaction between MPs and DBP was antagonistic at low concentrations of DBP Synergistic effect was found at relatively high concentrations of DBP when [MPs]< 10mg/L Antagonistic effect was found across all concentrations of MPs above 10 mg/L	[148]
Rigid PVC	4–141 µm	Diisononylphthalate (DiNP)	Rigid PVC (PVC) 4320 MP particles/100mL Flexible PVC with DiNP 4320 particles/100 mL ([DiNP] in PVC was 30% of plastic weight)	25–31 days	*Daphnia magna*	Mortality rate Morphology changes and body length Reproductive output	MPs containing DiNP significantly affect the number of offspring as well as the growth of D. magna The relevance of long-term chronic exposure experiments, as effects did emerge relatively late in the experiment	[149]
Flexible PVC	12–276 µm							

* Shadowed cells represent environmental relevant concentrations.

## Abbreviations:

4-MBC	4-Methylbenzylidene-camphor
AChE	Acetylcholinesterase
BPA	Bisphenol A
DDTs	Dichlorobiphenyl Trichloroethanes
DOM	Dissolved Organic Matter
EROD	7-ethoxyresorufin O-deethylase
GSH	Glutathione
HCHs	Hexachlorocyclohexanes
HDPE	High-Density Polyethylene
HOCs	Hydrophobic Organic Compounds
Kow	Octanol-water partition coefficient
Kpw	Plastic-water partition coefficient
LDPE	Low-Density Polyethylene
LPO	Lipid Peroxidation
MDA	Malondialdehyde
MPs	Microplastics
MTLP	Metallothionein-like Proteins
NOAA	US National Oceanographic and Atmospheric Administration
NPs	Nanoplastics
NSAID	Nonsteroidal Anti-inflammatory Drugs
OPFRs	Organophosphorus Flame-Retardants
PA	Polyamide
PAHs	Polycyclic Aromatic Hydrocarbons
PBDEs	Polybrominated Diphenyl Ethers
PCBs	Polychlorinated Biphenyls
PE	Polyethylene
PET	Polyethylene Terephthalate
PFAS	Perfluoroalkyl substances
PFNA	Perfluornonanoic Acid
PFOS	Perfluorooctanesulfonate
PFOSA	Perfluorooctanesulfonamide
PhACs	Pharmaceuticals Active Compounds
POPs	Persistent Organic Pollutants
PP	Polypropylene
PTFE	Polytetrafluoroethylene
PVC	Polyvinyl Chloride
SOD	Superoxide Dismutase
TBBPA	Tetrabromobisphenol A
Tg	Glass Transition Temperature
